# Testosterone is positively associated with coronary artery calcium in a low cardiovascular disease risk population

**DOI:** 10.1093/emph/eoad039

**Published:** 2023-11-16

**Authors:** Benjamin C Trumble, Jacob Negrey, Stephanie V Koebele, Randall C Thompson, L Samuel Wann, Adel H Allam, Bret Beheim, M Linda Sutherland, James D Sutherland, Daniel Eid Rodriguez, David E Michalik, Chris J Rowan, Guido P Lombardi, Angela R Garcia, Daniel K Cummings, Edmond Seabright, Sarah Alami, Thomas S Kraft, Paul Hooper, Kenneth Buetow, Andrei Irimia, Margaret Gatz, Jonathan Stieglitz, Michael D Gurven, Hillard Kaplan, Gregory S Thomas, Tsimane Health and Life History Project Teams

**Affiliations:** Arizona State University, School of Human Evolution and Social Change, Center for Evolution and Medicine, Institute of Human Origins, Tempe, AZ, USA; Arizona State University, School of Human Evolution and Social Change, Center for Evolution and Medicine, Institute of Human Origins, Tempe, AZ, USA; Arizona State University, School of Human Evolution and Social Change, Center for Evolution and Medicine, Institute of Human Origins, Tempe, AZ, USA; Saint Luke’s Mid America Heart Institute, Department of Cardiology, Kansas City, MO, USA; University of New Mexico, School of Medicine, Albuquerque, NM, USA; Al Azhar University, School of Medicine, Cairo, Egypt; Max Planck Institute for Evolutionary Anthropology, Department of Human Behavior, Ecology and Culture, Leipzig, Germany; MemorialCare Health System, Fountain Valley, CA, USA; MemorialCare Health System, Fountain Valley, CA, USA; Universidad de San Simón, Department of Medicine, Cochabamba, Bolivia; University of California Irvine, School of Medicine, Irvine, CA, USA; Miller Women’s and Children’s Hospital Long Beach, CA, USA; University of Nevada, School of Medicine, NV, USA; Universidad Peruana Cayetano Heredia, Laboratorio de Paleopatología, Lima, Peru; Arizona State University, School of Human Evolution and Social Change, Center for Evolution and Medicine, Institute of Human Origins, Tempe, AZ, USA; Chapman University, Economic Science Institute, Orange, CA, USA; Mohammed VI Polytechnic University, School of Collective Intelligence, Ben Guerir, Morocco; Mohammed VI Polytechnic University, School of Collective Intelligence, Ben Guerir, Morocco; University of Utah, Anthropology Department, Salt Lake City, UT, USA; Chapman University, Economic Science Institute, Orange, CA, USA; Arizona State University, School of Human Evolution and Social Change, Center for Evolution and Medicine, Institute of Human Origins, Tempe, AZ, USA; University of Southern California, Psychology Department, Los Angeles, CA, USA; University of Southern California, Psychology Department, Los Angeles, CA, USA; Toulouse Scool of Economics, Institute for Advanced Study Toulouse, Toulouse, France; University of California Santa Barbara, Department of Anthropology, Santa Barbara, CA, USA; Chapman University, Economic Science Institute, Orange, CA, USA; MemorialCare Health System, Fountain Valley, CA, USA; University of California Irvine, Division of Cardiology, Orange, CA, USA

**Keywords:** testosterone, cardiovascular disease, evolutionary mismatch, coronary artery calcium

## Abstract

**Background:**

In industrialized populations, low male testosterone is associated with higher rates of cardiovascular mortality. However, coronary risk factors like obesity impact both testosterone and cardiovascular outcomes. Here, we assess the role of endogenous testosterone on coronary artery calcium in an active subsistence population with relatively low testosterone levels, low cardiovascular risk and low coronary artery calcium scores.

**Methodology:**

In this cross-sectional community-based study, 719 Tsimane forager-horticulturalists in the Bolivian Amazon aged 40+ years underwent computed tomography (49.8% male, mean age 57.6 years).

**Results:**

Coronary artery calcium levels were low; 84.5% had no coronary artery calcium. Zero-inflated negative binomial models found testosterone was positively associated with coronary artery calcium for the full sample (Incidence Rate Ratio [IRR] = 1.477, 95% Confidence Interval [CI] 1.001–2.170, P = 0.031), and in a male-only subset (IRR = 1.532, 95% CI 0.993–2.360, P = 0.053). Testosterone was also positively associated with clinically relevant coronary atherosclerosis (calcium >100 Agatston units) in the full sample (Odds Ratio [OR] = 1.984, 95% CI 1.202–3.275, P = 0.007) and when limited to male-only sample (OR = 2.032, 95% CI 1.118–4.816, P = 0.024). Individuals with coronary artery calcium >100 had 20% higher levels of testosterone than those with calcium <100 (t = –3.201, P = 0.007).

**Conclusions and Implications:**

Among Tsimane, testosterone is positively associated with coronary artery calcium despite generally low normal testosterone levels, minimal atherosclerosis and rare cardiovascular disease (CVD) events. Associations between low testosterone and CVD events in industrialized populations are likely confounded by obesity and other lifestyle factors.

## INTRODUCTION

Testosterone plays an important role in male reproductive trade-offs across a number of species including humans. Testosterone is associated with energetically expensive muscle tissue [1–3], and during energetic stressors like illness or injury [4–6], high energetic expenditure [7], or reduced caloric intake [8, 9], testosterone declines rapidly within hours to days. Because of these energetic trade-offs, male participants in industrialized populations tend to have higher circulating testosterone than male age-matched subsistence populations with high parasite and pathogen loads [10–12]. However, in industrialized populations, increases in body fat with age result in the aromatization of testosterone to estradiol and declines in circulating testosterone with age [13]. As such, low endogenous male testosterone in industrialized populations is often associated with a higher risk of morbidity and all-cause mortality, including higher rates of cardiovascular disease (CVD) [14–21].

While low testosterone has been associated with cardiovascular morbidity [14, 15, 19–21], a *causal* relationship between low testosterone and atherosclerotic CVD has not been established [22] and remains controversial [18, 19]. Notably, most CVD data are drawn from industrialized populations that exhibit species-atypical (and perhaps maladaptive) lifestyles characterized by excessive sedentary inactivity [23, 24], diets high in saturated fats and in simple sugars, low in fiber [25, 26], with high rates of chronic disease [27]. The cumulative physiological disparities distinguishing industrialized from subsistence populations are reflected in testosterone levels: industrialized populations exhibit significantly higher testosterone levels in early adulthood than do members of small-scale societies [10–12]. Yet, despite elevated levels of testosterone across much of adulthood, industrialized populations exhibit higher rates of CVD and associated risk factors than do small-scale societies [28, 29]. Given divergent patterns of testosterone secretion and CVD prevalence across populations, observed associations between testosterone and atherosclerotic CVD in industrialized populations may reflect a species-atypical pattern that blurs or obscures any mechanistic relationships between testosterone and coronary artery disease (CAD) progression.

**Figure 1.**
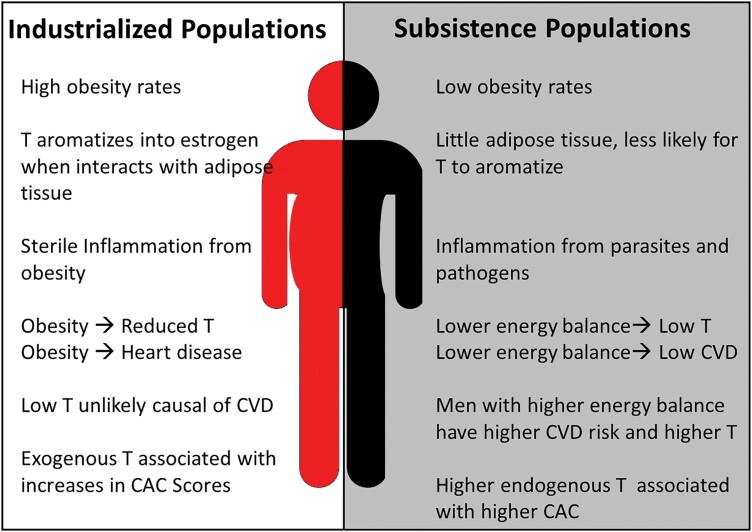
Conceptual figure highlighting differences in testosterone physiology and the associations between testosterone and CVD risk in industrial and subsistence populations.

Confounding factors such as obesity that are prevalent in industrialized societies make it difficult to isolate the impact of testosterone on CVD, as obesity is associated with both increased risk of CVD and low testosterone [13, 30]. In the Multiethnic Study of Atherosclerosis (MESA), lower male testosterone was associated with higher rates of hypertension, diabetes, higher body mass index and higher rates of atherosclerosis as measured by coronary artery calcium (CAC) [31]; see Fig. 1. Males in industrial populations with many chronic diseases from cancer, hyperuricemia, T2DM, high inflammation and CVD have lower circulating testosterone [14, 15, 18–20, 22, 32–36]. As these studies are observational in nature, it is not possible to assess causality, but it should be noted that there is also the potential for reverse causality; inflammatory processes that are involved in CVD and many chronic diseases can downregulate testosterone production [5, 6]. Adipose tissue is a significant source of inflammation in industrialized populations [37], sometimes referred to as sterile inflammation (as opposed to infectious-based inflammation [38]), and thus could present a multifactorial impact decreasing testosterone production, as well as increasing CVD risk [39].

While observational studies suggest that lower testosterone is associated with CVD, testosterone supplementation showed little benefit [40] or even increased CAC following treatment [41]. Commonly reported associations between testosterone and CVD in relatively sedentary obese industrialized populations may be additionally confounded by lifestyle factors like diet or physical activity, where obesity is typically correlated with both low testosterone and CVD [13] (Fig. 1). While coronary atherosclerosis begins as a non-calcified plaque in the arterial wall, as a plaque matures calcium hydroxyapatite is deposited in the plaque and its presence in a coronary artery is diagnostic of atherosclerosis [42, 43]. Measurement of CAC by chest computed tomography scanning is often used to detect pre-clinical CAD and is a tool to assess the burden of coronary atherosclerosis [44]. Furthermore, CAC is correlated with coronary atherosclerotic burden, and is predictive of future cardiovascular events and all-cause mortality [45, 46].

Here, we test for a testosterone- CAD association in a physically active population with few CAD risk factors, but relatively low testosterone, compared to age-matched US males [47]. The Tsimane, a remote population of forager-horticulturalists in the Bolivian Amazon [48], show levels of testosterone that are generally in the low-to-normal range by US standards across their lifespan, with testosterone 37.3% lower than age-matched US males [47]. Unlike documented patterns in the USA and other high-income countries, male Tsimane displays relatively minimal age-related declines in testosterone [49], perhaps due to low body fat deposition with age [50], which leads to lower testosterone in US males [18]. Body fat can also influence many immune processes, including inflammation, which can lead to lower testosterone; thus, we also examine associations between cytokines, body fat and testosterone. The Tsimane practice a physically intensive, traditional subsistence lifestyle based on small-scale horticulture, fishing, hunting and gathering [48], and are one of the most heart-healthy populations ever examined clinically, with minimal obesity and hypertension, low blood lipids, negligible atrial fibrillation and minimal CAC [51, 52].

## METHODS

### Coronary artery calcium

Tsimane adults (*n* = 719, 49.8% male) aged 40–94 years underwent thoracic non-contrast CT scanning using a 16-detector row, multi-slice scanner (GE Brightspeed, Milwaukee, WI). A licensed radiology technician acquired electrocardiogram-gated CT scans. All scans were supervised and reviewed by HORUS team cardiologists and radiologists. Calcium in the coronary arteries was scored using Siemens (Erlangen, Germany) Syngo.via calcium scoring semi-automatic software, which was then reviewed by a technician blind to participant data. Candidate lesions were reviewed and scored [53] using protocols similar to those employed in large population samples [54].

Since 2010, the Tsimane Health and Life History Project (THLHP) has conducted continuous epidemiological surveillance on >85% of the Tsimane population. Among adults over 40 years of age, clinical exams, biomarkers, demographic information and other interviews are collected annually [48]. Body fat is measured via bioelectric impedance (Tanita BC-1500), and age is verified through a multi-step process involving known dated events, birth cohort-rankings, demographic histories and census records [48]. For this study, 719 individuals were sampled from 1214 who met the inclusion criteria (40+ years of age and self-identifying as Tsimane). Age stratification of the sample ensures even decile representation, requiring sampling of all available individuals ≥60 years and a random subset of individuals 40–59 years [51]. Individuals over age 60, not sampled had either recently migrated following major flooding (*n* = 49), were presently engaged in horticultural labor or hunting (*n* = 15), or refused to participate (*n* = 2). Only one individual refused to participate because of poor health or inability to travel (recovering from a hernia surgery). To address potential bias, those who had computed tomography scans (CTs) were compared to those without CTs; there were no significant differences in sex (*P* = 0.634), systolic (*P* = 0.301) or diastolic (*P* = 0.301) blood pressure, or body fat (*P* = 0.942), indicating a representative sample of adults over 40 years of age.

### Blood lipids and testosterone and immune markers

Fasting morning blood was drawn shortly after waking (6–8 am), and serum was stored in liquid nitrogen until transferred to the laboratory. Lipids were measured (Stat Fax 1908, Awareness Technology, Palm City, FL, USA) in the THLHP laboratory in San Borja, Beni, Bolivia, and testosterone and cytokines were measured in singlicate via enzyme immunoassay in the University of California Santa Barbara Biodemography lab [49]. Within (intra plate) and between (inter) plate coefficients of variation were 5.9% and 14.9% for the high controls, and 6.3% and 11.7% for the low controls, which were run in duplicate on all plates. Cytokines (interleukin [IL]-1B, IL-2, IL-4, IL-5, IL-6, IL-10, IL-13, granulocyte-macrophage colony-stimulating factor and interferon [IFN]-gamma INFG) were measured via Luminex multiplex (EMD Millipore, Darmstadt, Germany). Following the manufacturer's recommendation, calibrators were prepared with a serum matrix, and specimens below the limits of detection were assigned the lower limit of detection. Quality control specimens were within the expected ranges provided by the manufacturer, see Ref. [51].

### Statistical approach

To evaluate the association between testosterone and CAC scores while adjusting for known covariates of Tsimane CAC [51], we performed a multivariate zero-inflated negative binomial regression [55] to account for the large fraction of ‘zero’ CAC scores and over-dispersion of data. The model performs a logistic regression to test for factors that predict excess CAC absence, and a simultaneous negative binomial regression to examine factors associated with CAC scores (including zeros and positive scores). CAC >100 is considered moderate, clinically relevant atherosclerosis [51, 54]; additional logistic regressions examined the association between CAC >100 and testosterone with relevant control variables, but see limitations. Testosterone was log transformed for normality; other variables had normal distributions. Models were selected by Akaike’s Information Criteria, starting from the base model from previously published work on Tsimane CAC [51]. Males and females differ in testosterone level and variation as well as CAC, resulting in significant heteroscedasticity (unequal variance across sex), necessitating separate analyses for males and females. It should be noted that testosterone had more explanatory value in explaining differences in CAC score than biological sex. US studies focus only on males so it is not possible to examine sex differences in the effects of testosterone across populations.

### Institutional review board approval

Informed consent was collected at three levels: by the individual, by the community and by the Tsimane Gran Consejo (Tsimane governing body). The procedures were approved by the institutional review boards at the University of California, Santa Barbara and University of New Mexico, and Universidad San Simon Mayor, Cochabamba Bolivia.

### Role of the funding source

This study was funded by the National Institute of Health, National Institute on Aging (NIH/NIA) 1R01-AG054442 and R01-AG024119. The funding organization played no role in the study design, methods, or analyses.

## RESULTS

### Risk factors

The Tsimane are a relatively lean population with low atherosclerotic risk factors, and in this sample of 719 adults, there are low levels of CAC through the eighth decade of life (Table 1). Despite no statin use, 0% of male participants, and only 0.9% of female participants had elevated total cholesterol (>240 mg/dL) while 13.8% of male and 15.6% of female participants had LDL greater than 130 mg/dL. HDL is relatively low in this population with 53.2% of male and 49.4% of female participants below 40 mg/dL. Obesity is unusual with only 3.3% of male and 8.8% of female Tsimane presenting with BMI >30 kg/m^2^. Previous studies report that testosterone is significantly lower (37.3% lower) among the Tsimane compared to aged-matched US males [47], and has a shallower rate of decline with age [47, 49].

**Table 1.** Biometrics and CVD risk factors by sex for *n* = 719 Tsimane

Biomarker (mean)	Male	Male SD	Female	Female SD	*P* value
Coronary Artery Calcium score (AU)	11.3	43.1	6.9	57.1	0.239
Age, years	57.2	10.3	58.0	10.7	0.302
BMI, kg/m^2^	24.1	2.9	24.0	4.0	0.777
Body fat, %	18.2	6.2	26.0	7.9	**<0.001**
Systolic BP, mmHg	117.3	12.4	115.0	12.7	**0.015**
Diastolic BP, mmHg	74.9	10.2	72.3	9.9	**<0.001**
Total testosterone, ng/dL	779.9	1034.1	297.8	370.6	**<0.001**
IL-5, pg/mL	2.5	2.5	3.0	6.2	0.145
IL-10, pg/mL	4.8	5.6	4.2	6.8	0.251
Cholesterol, mg/dL	148.7	31.8	152.4	28.9	0.105
LDL, mg/dL	96.0	31.0	101.8	30.5	**0.016**
HDL, mg/dL	40.2	8.1	38.8	7.4	**0.017**
Triglycerides, mg/dL	106.0	43.3	98.6	47.2	**0.031**
Glucose, mg/dL	79.1	11.8	78.9	10.3	0.900
% High LDL (>130 mg/dL)	13.8%		15.6%		0.484
% High cholesterol (>240 mg/dL)	0.0%		0.9%		0.083
% High triglycerides (>200 mg/dL)	3.3%		4.3%		0.752
% Low HDL (<40 mg/dL)	52.1%		57.6%		0.170
% High glucose (>125 mg/dL)	0.6%		0.0%		0.158
% Obese BMI (>30 kg/m^2^)	3.3%		8.8%		**0.002**
% Hypertensive (Systolic >140 or Diastolic >90 mmHg)	6.2%		4.7%		0.484

AU, Agatston units; BMI, body mass index; BP, blood pressure; HDL, high-density lipoprotein; IL, interleukin; LDL, low-density lipoprotein; SD, standard deviation. Bold indicates statistically significant at *P* < 0.05.

### Low levels of coronary artery calcium

As previously reported [51], the Tsimane have the lowest known levels of CAC for any population to date (Table 1). Only 15.5% of Tsimane presented with any CAC, while comparative samples of MESA data show more than 86% of Americans in the same age range have CAC. Similarly, only 2.7% of Tsimane had CAC scores >100 Agatston units (AU), while more than 50% of MESA participants had calcium scores >100 AU [56].

### Testosterone and CAC

**Figure 2.**
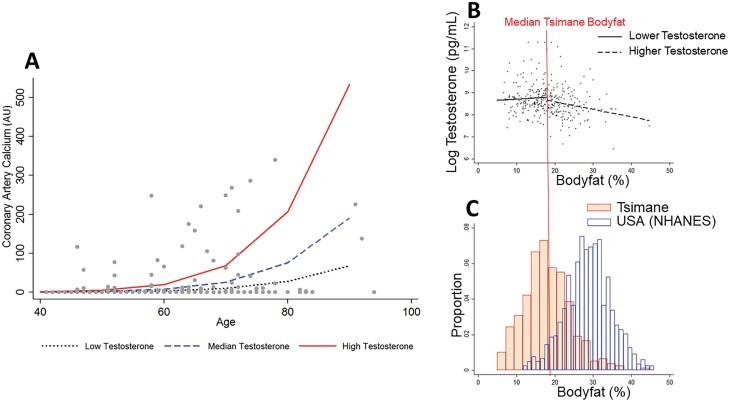
(A) Male CAC by age and tertile of testosterone. (B) Associations between testosterone and body fat differ for male Tsimane above and below median body fat. (C) Tsimane males have low body fat compared to age-matched US males (NHANES). Only 3.3% of US males fall below the median Tsimane body fat, and thus nearly all US males fall on the portion of the regression line where higher body fat is associated with low testosterone.

While CAC levels are extremely low in this population, higher testosterone was associated with higher CAC values for male and female participants who had CAC (IRR = 1.474, *P* = 0.049, Table 2; Fig. 2a). There was no interaction between sex and testosterone on overall CAC extent, though males were more likely to have non-zero CAC. Male sex, older age, HDL and triglycerides all positively predicted CAC presence, while higher testosterone, age, body fat and lower IL-10 were associated with continuous CAC levels above zero (Table 2). When the model was limited to males only, testosterone did not significantly associate with the probability of presenting with CAC, but there was a positive trend between testosterone levels and the extent of CAC for those who had CAC (IRR = 1.532, *P* = 0.054, Table 2). Age, HDL and triglycerides all positively predicted CAC presence in male Tsimane, while age and IL-10 were associated with CAC extent (Table 2). A female-only subset found no significant effect of testosterone on CAC.

**Table 2.** Associations between testosterone and CAC

A.Zero-inflated negative binomial model predicting Tsimane CAC scores by log testosterone for both sexes together and individually												
	IRR	*P* value	95% Conf. Int		IRR	*P* value	95% Conf. Int		IRR	*P* value	95% Conf. Int	
Predictor of CAC score, both sexes					Male only				Female only			
Log testosterone (pg/mL)	1.474	0.049	1.001	2.170	1.532	0.054	0.993	2.360	0.938	0.902	0.338	2.604
Age (years)	1.070	<0.001	1.032	1.109	1.071	0.002	1.026	1.117	1.047	0.127	0.987	1.110
Body fat (%)	1.060	0.017	1.011	1.112	1.015	0.685	0.945	1.090	1.121	0.020	1.018	1.234
IL-5 (pg/mL)	0.848	0.167	0.670	1.072	1.113	0.538	0.792	1.564	0.624	0.005	0.448	0.870
IL-10 (pg/mL)	0.924	0.042	0.856	0.997	0.897	0.010	0.826	0.974	0.950	0.637	0.768	1.175
Intercept	0.010	0.043	0.000	0.869	0.010	0.109	0.000	2.774	0.398	0.843	0.000	3521.9
Predictor of CAC absence (zero Inflation)												
Male	–1.243	<0.001	–1.841	–0.645								
Age (years)	–0.071	<0.001	–0.098	––0.045	–0.083	<0.001	–0.120	–0.045	–0.060	0.002	–0.098	–0.022
HDL (mg/dL)	–0.029	0.105	–0.063	0.006	–0.043	0.049	–0.085	0.000	–0.0001	0.996	–0.061	0.601
Triglycerides (mg/dL)	–0.009	0.007	–0.016	–0.003	–0.009	0.077	–0.019	0.001	–0.009	0.056	–0.018	0.0002
Intercept	8.434	0.000	5.987	10.880	8.493	<0.001	5.378	11.608	6.514	0.001	2.824	10.203

Zero-inflated negative binomial model predicting Tsimane CAC scores by log testosterone for both sexes.

AU, Agatston units; CAC, coronary artery calcium; HDL, high-density lipoprotein; IL, interleukin.

### Testosterone and clinically relevant atherosclerosis

In addition to its association with subclinical CAC, testosterone was associated with a higher likelihood of having CAC >100 for both the full sample (OR = 1.984, *P* = 0.007, Pseudo *R*^2^ = 0.156, Table 3), and when the sample was limited to males only (OR = 2.320, *P* = 0.024, Pseudo *R*^2^ = 0.1932, Table 3). Males and females with CAC >100 had significantly higher testosterone than those without high CAC (median testosterone with CAC >100 = 417 ng/dL, median testosterone with CAC <100 = 341 ng/dL; *t* = –3.201, *P* = 0.0007).

**Table 3.** Logistic regression models testing association between log testosterone and clinically relevant atherosclerosis (CAC >100 AU)

CAC >100 AU both sexes					CAC >100 AU male only				CAC >100 AU female only			
	OR	*P* value	95% Conf. Int		OR	*P* value	95% Conf. Int		OR	*P* value	95% Conf. Int	
Log testosterone (pg/mL)	1.984	0.007	1.202	3.275	2.320	0.024	1.118	4.816	1.212	0.700	0.456	3.220
Age (years)	1.010	<0.001	1.056	1.138	1.114	<0.001	1.060	1.171	1.076	0.022	1.011	1.145

### Testosterone and CAD risk factors

Both male and female Tsimane generally showed low levels of classical CVD risk factors (Table 1). However, testosterone had a non-linear association with one CAD risk factor-body fat. Controlling for age, Tsimane male body fat was positively (though not significantly) associated with testosterone for those below the median body fat (17.6%), and negatively associated with testosterone for those above median body fat (*β* = –0.05, *P* = 0.002), Fig. 2B. Age matched representative data from US males (NHANES) show significantly higher male body fat; the median US male has a body fat of 28.9%, with only 3.3% of US males falling below the Tsimane median (Fig. 2C). In female Tsimane, testosterone was not associated with body fat or any other traditional risk factors.

### Inflammation, testosterone and body fat

In a combined model with both sexes, those with higher log IL-2 had significantly lower levels of log testosterone (*β* = –0.13, *P* = 0.002) controlling for age, sex and body fat, following Bonferroni correction (see Supplementary Table S1). Body fat was not significantly associated with any cytokines in the full model.

In the male-only subset, those with higher log IL-2 and log IL-5 had lower log testosterone, but these results were not statistically significant following Bonferroni correction, (see Supplementary Table S2). Higher levels of log IL-1b were associated with higher body fat controlling for age and testosterone, but these results were not statistically significant following Bonferroni correction.

### Comparison to clinical cutoffs

Various clinical cutoffs for ‘low’ male testosterone or clinical hypogonadism have been used in the past in industrialized populations [57]. MESA studies used cutoffs of 300 ng/dL and 275 ng/dL, finding that US males below either of these cutoffs showed increased CAC [31]. For male Tsimane, having testosterone lower than these cutoffs was not associated with increased CAC (14.2% of male Tsimane in this sample had testosterone <300 ng/dL). On the contrary, participants below either of the MESA cutoffs had less CAC than those above the cutoffs. Similarly, male Tsimane below the clinical cutoff of hypogonadism of 230 ng/dL also had lower CAC scores than those with higher CAC [31]. Table 4 shows CAC scores by tercile for Tsimane testosterone.

**Table 4.** Testosterone in males and CAC score by testosterone tercile (low, middle, high)

	Low tercile *T n* = 114	Middle tercile *T n* = 114	High tercile *T n* = 115
Median testosterone ng/dL	322.7	513.0	931.4
Median age	60	52	52.5
Mean CAC	7.5	13.7	3.6
% CAC > 0 AU	27.4%	13.4%	21.4%
% CAC > 100 AU	4.4%	2.6%	5.3%

AU, Agatston units; CAC, coronary artery calcium.

## DISCUSSION

The Tsimane population offers a unique test of the association between endogenous testosterone and coronary calcium with low levels of confounding CVD risk factors like obesity seen in industrialized populations. While no single population is a perfect exemplar of the myriad of environments in which humans evolved, sedentary urban environments are evolutionarily novel and not a good representation of what healthy aging was like prior to the last several hundred years. Most medical research is conducted in these sedentary environments, and thus our understanding of associations between cardiovascular disease and testosterone may be skewed. By working with populations that still have a physically active subsistence life style, we can better understand how human physiology is associated with healthy aging, without the confounding of obesity and other related factors. Though studies from industrial urban centers like MESA report higher coronary calcium in male participants with low testosterone [31], we find that male Tsimane with higher testosterone have *more* coronary calcium, while there is no relationship between testosterone and coronary calcium in female Tsimane participants (note: MESA investigators did not describe associations between testosterone in female participants and CAC) [7]. While high levels of CAC indicative of clinically relevant atherosclerosis are roughly 10-fold lower among Tsimane than in the USA [51], Tsimane with higher circulating testosterone were 2.5 times more likely to exhibit CAC scores over 100 than Tsimane with low testosterone.

Despite having one-third lower levels of testosterone than age-matched US males, even Tsimane with the lowest levels of testosterone were not at risk of atherosclerosis. In fact, not only does Tsimane have low levels of testosterone and low CVD risk, but Tsimane with higher levels of testosterone actually present with higher levels of atherosclerosis, and have a higher risk of clinically relevant CAC scores. These results call into question whether low testosterone plays a causal role in atherosclerosis, or whether the association between low testosterone and CVD is indirect. While male participants in industrial populations with low testosterone often are obese or have other cardiovascular risk factors such as diabetes [18, 30, 31], low testosterone was not associated with traditional cardiovascular risk factors in the Tsimane. The complex and often inconsistent results available in the testosterone and cardiovascular morbidity literature may stem from heterogeneity in underlying levels of CVD risk factors. This study is unique in that the Tsimane show exceptionally low levels of CVD risk factors, making it an ideal population in which to measure the effects of testosterone on CAD without confounding other risk factors.

Aromatization of testosterone by adipose tissue has been linked to the lower circulating levels of testosterone in obese male participants in industrialized populations [13], and obesity can alter the gut microbiome in ways that impact steroid hormone concentrations as well [58]. However, males in industrialized populations have significantly higher body fat (median US male body fat is 48.6% higher than median Tsimane body fat). We speculate that if US males fell within the range of body fat observed in Tsimane males, we would not see a purely detrimental impact of body fat on testosterone (Fig. 2B and C). Indeed, among male Olympians, androgen levels were significantly lower in athletes considered ‘lean’ (body fat 11.7% SD ± 3.4%) versus ‘nonlean’ (body fat 16.4% SD ± 5.8%) [59]. Individuals in a depleted energetic condition are likely to have downregulated testosterone and low body fat. Those in better energetic condition can maintain higher testosterone and higher body fat, but once body fat levels get above a threshold, testosterone will begin to decrease as it is aromatized into estradiol. Among the Tsimane, high body fat is negatively associated with testosterone while there is no association between testosterone and below median levels of body fat. Nearly all US males have high body fat by Tsimane standards, which may help explain why testosterone is consistently negatively associated with body fat in industrialized populations (Fig. 2B and C). The relatively low levels of testosterone among Tsimane may instead be due to their high pathogen burden and low food security as a result of energetic tradeoffs [48, 60]. It is physiologically expensive to maintain high levels of testosterone, and males experiencing illness [5], injury [6] or sustained caloric deficiency show rapid, substantive declines in testosterone [7]. Similarly, low levels of testosterone have been reported among other subsistence populations facing high pathogen burden [11]. Notably, due to relatively low testosterone, Tsimane have relatively small prostate volumes compared to other populations with higher testosterone, resulting in some of the lowest population levels of benign prostatic hyperplasia [49]. Yet, similar to what is reported in the USA, we find a positive association between testosterone and cognitive function among the Tsimane [61]. We suspect that in these populations and in similar contexts, lower testosterone may also be associated with lower, not greater, atherosclerotic burden.

We did not find strong associations between inflammation and testosterone in this sample. Of nine cytokines measured, only IL-2 was significantly associated with testosterone, with higher IL-2 predicting lower levels of testosterone. This finding fits with the experimental literature where IL-2 infusions decrease testosterone production in human males in industrialized populations [62]. While higher levels of inflammation are often associated with low testosterone in industrialized populations, we do not find the same associations here, nor do we see that higher adiposity is associated with higher inflammation [37]. The lack of association between inflammation and body fat may be because of differences in the type of inflammation; while individuals in industrialized populations experience sterile inflammation from obesity, smoking and pollution, the Tsimane are experiencing high inflammation from parasites and pathogens [37, 38, 60].

To date, the causal role of testosterone in cardiovascular health has not been well established [63]; and it should be noted that we are only reporting relationships in this study and not causal associations. Studies have reported associations between low testosterone and CVD risk factors, cardiovascular disease rates (as measured by CAC), percutaneous coronary intervention-related major adverse cardiac events, and all-cause mortality [14, 15, 18–21, 31]. There are several biologically plausible mechanisms by which exogenous testosterone could be cardioprotective either directly or by reducing CVD risk factors [22, 64]; exogenous testosterone increases exercise tolerance in males with stable angina [64], increases coronary blood flow [65], and cardiac output [65, 66], and is associated with decreased reperfusion injury [66]. However, testosterone therapy may come at a health risk; it is associated with an increase in myocardial infarction [67, 68], increased risk of thrombosis [69], and higher rates of stroke in most [70], though not all, studies [71]. Testosterone administration is associated with an increase in total plaque volume but does not appear to impact CAC deposition over a one-to-three-year period [40, 41]. Thus, testosterone administration may have both harmful effects with an increased rate of plaque formation and thrombosis risk as well as positive effects on arterial elasticity and cardiac perfusion [22, 68, 72, 73].

This creates an apparent contradiction where low endogenous testosterone is associated with higher rates of CAD [14, 15, 18–21, 31], but exogenous testosterone can also have deleterious cardiovascular effects [40, 41, 67–70]. This may be explained by the underlying cause of low endogenous testosterone in males with CVD: males with higher body fat have lower testosterone due to aromatization to estradiol [13]. Thus, obesity and its related disease states, not circulating testosterone, are likely responsible for poor CVD outcomes in males with low testosterone. In the USA, males taking supplemental testosterone can maintain both high body fat and high testosterone, an unnatural and potentially risk-inducing combination if both testosterone and obesity independently contribute to CVD.

These contradictory impacts of testosterone on cardiovascular risk could explain why the testosterone supplementation literature shows some positive impacts of testosterone [22, 64], despite overall negative impacts of testosterone supplementation on cardiovascular morbidity and mortality [68, 73]. There has been a large increase in the number of prescriptions for testosterone supplementation over the last decade: nearly a 10-fold increase in the USA and 100-fold increase worldwide [63, 74]. While existing data may not yet be sufficient to assess the effects of testosterone on CVD, new data from the large numbers of US males now taking exogenous testosterone may illuminate additional pathways and mechanisms.

## Limitations

Most medical research is conducted in sedentary urban populations, and while this study adds a very underrepresented type of population, there are limits to the generalizability of these results. While the Tsimane have relatively low CVD risk factors, and while that allows us to examine associations between testosterone and CVD without certain confounders, the high parasite and pathogen burden [60], and differential genetic structure of the population [75] may limit cross-population comparisons. A relatively small proportion of this sample had CAC scores over 100 AU (2.7% of the total sample, *n* = 20 individuals, including 14 male and 6 female participants). Thus, these results (Table 3) should be interpreted with caution. Due to limitations in sample volume and cost, testosterone and cytokines were run in singlicate, though assay standards and controls and run in duplicate to assess assay functionality. This could potentially add additional random variation to the sample.

We used a single testosterone measurement, which may not be indicative of an individual’s entire lifetime exposure to testosterone, as testosterone can fluctuate rapidly under multiple types of stressors [5–8]. That said, testosterone is relatively stable with age in this population [76, 77], and specimens collected 2 years apart (*n* = 135) had an intraclass correlation (ICC) of 0.45, indicating relatively fair stability across time points with approximately half of the variability due to differences between individuals. Our use of single-point measures of testosterone is comparable to what is reported in other studies in industrialized populations [14–16, 19, 20, 31]. We measured total testosterone, but not sex hormone binding globulin or albumin, and thus cannot assess how much of that testosterone was biologically available or bound to carrier proteins. Enzyme immunoassays designed to measure just free testosterone are not considered accurate [78], and the Endocrine Society position statement recommends serum total testosterone measurements for both males and females [78]. Total testosterone has been measured in other testosterone-CVD studies in industrialized populations [14–16, 19, 20, 31].

## CONCLUSIONS

While prior studies have shown that low levels of serum testosterone are associated with increased rates of coronary artery calcification, we found the opposite effect in a population with very low rates of atherosclerosis risk factors and low rates of CAC. Our findings suggest that the previously described association between CAC and low testosterone is indirect rather than causal.

## Supplementary Material

eoad039_suppl_Supplementary_Tables_S1

eoad039_suppl_Supplementary_Tables_S2

## Data Availability

Individual-level data are stored in the Tsimane Health and Life History Project (THLHP) Data Repository and are available through restricted access for ethical reasons. THLHP’s highest priority is the safeguarding of human subjects and minimization of risk to study participants. The THLHP adheres to the ‘CARE Principles for Indigenous Data Governance’ (Collective Benefit, Authority to Control, Responsibility, and Ethics), which assure that the Tsimane (i) have sovereignty over how data are shared, (ii) are the primary gatekeepers determining ethical use, (iii) are actively engaged in the data generation and (iv) derive benefit from data generated and shared for use whenever possible. The THLHP is also committed to the ‘FAIR Guiding Principles for scientific data management and stewardship’ (Findable, Accessible, Interoperable, Reusable). Requests for individual-level data should take the form of an application that details the exact uses of the data and the research questions to be addressed, procedures that will be employed for data security and individual privacy, potential benefits to the study communities, and procedures for assessing and minimizing stigmatizing interpretations of the research results (see the following webpage for links to the data sharing policy and data request forms: https://tsimane.anth.ucsb.edu/data.html). Requests for individual-level data will require institutional IRB approval (even if exempt) and will be reviewed by an Advisory Council composed of Tsimane community leaders, community members, Bolivian scientists, and the THLHP leadership. The study authors and the THLHP leadership are committed to open science and are available to assist interested investigators in preparing data access requests.
