# Supplementary material for: Testosterone is positively associated with coronary artery calcium in a low cardiovascular disease risk population
Source: Evol Med Public Health. 2023 Nov 16;11(1):472–84. doi: 10.1093/emph/eoad039 (PMC10746324; doi:10.1093/emph/eoad039)
Supplement: eoad039_suppl_Supplementary_Tables_S1 [file eoad039_suppl_supplementary_tables_s1.docx]

Table S1: Regression models examining associations between each cytokine, body fat, age, sex, and testosterone.

| Log IL-1b | Coefficient | p-value | 95% CI | |
| --- | --- | --- | --- | --- |
| Body fat | 0.005 | 0.543 | -0.011 | 0.022 |
| Age | 0.004 | 0.482 | -0.007 | 0.015 |
| Male | 0.100 | 0.522 | -0.206 | 0.405 |
| Log Testosterone | -0.104 | 0.237 | -0.276 | 0.069 |
| Constant | 0.459 | 0.590 | -1.215 | 2.134 |

| Log IL-2 | Coefficient | p-value | 95% CI | |
| --- | --- | --- | --- | --- |
| Body fat | -0.006 | 0.129 | -0.014 | 0.002 |
| Age | 0.000 | 0.957 | -0.006 | 0.005 |
| Male | 0.067 | 0.390 | -0.085 | 0.219 |
| Log Testosterone | -0.134 | 0.002 | -0.220 | -0.049 |
| Constant | 2.137 | 0.000 | 1.305 | 2.968 |

| Log IL-4 | Coefficient | p-value | 95% CI | |
| --- | --- | --- | --- | --- |
| Body fat | -0.001 | 0.597 | -0.006 | 0.003 |
| Age | 0.001 | 0.476 | -0.002 | 0.004 |
| Male | -0.048 | 0.279 | -0.135 | 0.039 |
| Log Testosterone | 0.023 | 0.367 | -0.027 | 0.072 |
| Constant | 0.956 | 0.000 | 0.479 | 1.432 |

| Log IL-5 | Coefficient | p-value | 95% CI | |
| --- | --- | --- | --- | --- |
| Body fat | 0.006 | 0.358 | -0.007 | 0.018 |
| Age | 0.001 | 0.754 | -0.007 | 0.010 |
| Male | 0.172 | 0.145 | -0.060 | 0.404 |
| Log Testosterone | -0.116 | 0.080 | -0.247 | 0.014 |
| Constant | 1.144 | 0.077 | -0.126 | 2.413 |

| Log IL-6 | Coefficient | p-value | 95% CI | |
| --- | --- | --- | --- | --- |
| Body fat | -0.009 | 0.148 | -0.022 | 0.003 |
| Age | 0.012 | 0.007 | 0.003 | 0.021 |
| Male | 0.141 | 0.246 | -0.097 | 0.380 |
| Log Testosterone | -0.089 | 0.190 | -0.222 | 0.044 |
| Constant | 0.895 | 0.177 | -0.406 | 2.197 |

| Log IL-10 | Coefficient | p-value | 95% CI | |
| --- | --- | --- | --- | --- |
| Body fat | -0.004 | 0.475 | -0.014 | 0.007 |
| Age | 0.007 | 0.070 | -0.001 | 0.014 |
| Male | 0.121 | 0.216 | -0.071 | 0.314 |
| Log Testosterone | -0.098 | 0.077 | -0.206 | 0.011 |
| Constant | 1.550 | 0.004 | 0.498 | 2.602 |

| Log IL-13 | Coefficient | p-value | 95% CI | |
| --- | --- | --- | --- | --- |
| Body fat | 0.001 | 0.809 | -0.007 | 0.009 |
| Age | 0.006 | 0.031 | 0.001 | 0.011 |
| Male | -0.033 | 0.667 | -0.181 | 0.116 |
| Log Testosterone | 0.090 | 0.034 | 0.007 | 0.174 |
| Constant | -0.059 | 0.887 | -0.872 | 0.755 |

| Log GMCSF | Coefficient | p-value | 95% CI | |
| --- | --- | --- | --- | --- |
| Body fat | -0.007 | 0.185 | -0.017 | 0.003 |
| Age | 0.008 | 0.034 | 0.001 | 0.015 |
| Male | -0.014 | 0.883 | -0.207 | 0.178 |
| Log Testosterone | 0.000 | 0.996 | -0.108 | 0.109 |
| Constant | 0.654 | 0.222 | -0.398 | 1.706 |

| Log INFG | Coefficient | p-value | 95% CI | |
| --- | --- | --- | --- | --- |
| Body fat | 0.001 | 0.876 | -0.012 | 0.014 |
| Age | 0.002 | 0.621 | -0.007 | 0.011 |
| Male | 0.157 | 0.213 | -0.091 | 0.405 |
| Log Testosterone | -0.025 | 0.722 | -0.165 | 0.114 |
| Constant | 0.499 | 0.469 | -0.855 | 1.853 |
