# Supplementary material for: Testosterone is positively associated with coronary artery calcium in a low cardiovascular disease risk population
Source: Evol Med Public Health. 2023 Nov 16;11(1):472–84. doi: 10.1093/emph/eoad039 (PMC10746324; doi:10.1093/emph/eoad039)
Supplement: eoad039_suppl_Supplementary_Tables_S2 [file eoad039_suppl_supplementary_tables_s2.docx]

Table S2: Male only regression models examining associations between each cytokine, body fat, age, and testosterone.

| Log IL-1b | Coefficient | p-value | 95% CI | |
| --- | --- | --- | --- | --- |
| Body fat | 0.041 | 0.007 | 0.011 | 0.070 |
| Age | -0.005 | 0.557 | -0.022 | 0.012 |
| Log Testosterone | -0.125 | 0.362 | -0.393 | 0.144 |
| Constant | 0.627 | 0.663 | -2.204 | 3.458 |

| Log IL-2 | Coefficient | p-value | 95% CI | |
| --- | --- | --- | --- | --- |
| Body fat | 0.005 | 0.479 | -0.009 | 0.020 |
| Age | -0.005 | 0.297 | -0.013 | 0.004 |
| Log Testosterone | -0.166 | 0.016 | -0.300 | -0.032 |
| Constant | 2.525 | 0.001 | 1.110 | 3.940 |

| Log IL-4 | Coefficient | p-value | 95% CI | |
| --- | --- | --- | --- | --- |
| Body fat | 0.002 | 0.739 | -0.009 | 0.012 |
| Age | 0.003 | 0.328 | -0.003 | 0.009 |
| Log Testosterone | 0.056 | 0.262 | -0.042 | 0.153 |
| Constant | 0.456 | 0.382 | -0.569 | 1.482 |

| Log IL-5 | Coefficient | p-value | 95% CI | |
| --- | --- | --- | --- | --- |
| Body fat | 0.012 | 0.237 | -0.008 | 0.032 |
| Age | -0.005 | 0.380 | -0.017 | 0.007 |
| Log Testosterone | -0.221 | 0.019 | -0.406 | -0.036 |
| Constant | 2.491 | 0.012 | 0.543 | 4.440 |

| Log IL-6 | Coefficient | p-value | 95% CI | |
| --- | --- | --- | --- | --- |
| Body fat | 0.002 | 0.852 | -0.020 | 0.024 |
| Age | 0.005 | 0.435 | -0.008 | 0.018 |
| Log Testosterone | -0.111 | 0.264 | -0.307 | 0.084 |
| Constant | 1.425 | 0.179 | -0.655 | 3.505 |

| Log IL-10 | Coefficient | p-value | 95% CI | |
| --- | --- | --- | --- | --- |
| Body fat | -0.009 | 0.375 | -0.029 | 0.011 |
| Age | 0.008 | 0.181 | -0.004 | 0.020 |
| Log Testosterone | -0.092 | 0.330 | -0.278 | 0.094 |
| Constant | 1.625 | 0.104 | -0.333 | 3.583 |

| Log IL-13 | Coefficient | p-value | 95% CI | |
| --- | --- | --- | --- | --- |
| Body fat | 0.003 | 0.651 | -0.010 | 0.016 |
| Age | 0.002 | 0.606 | -0.006 | 0.010 |
| Log Testosterone | 0.025 | 0.674 | -0.092 | 0.142 |
| Constant | 0.667 | 0.290 | -0.572 | 1.905 |

| Log GMCSF | Coefficient | p-value | 95% CI | |
| --- | --- | --- | --- | --- |
| Body fat | -0.002 | 0.809 | -0.021 | 0.016 |
| Age | 0.011 | 0.062 | -0.001 | 0.022 |
| Log Testosterone | 0.081 | 0.352 | -0.090 | 0.251 |
| Constant | -0.301 | 0.741 | -2.094 | 1.492 |

| Log INFG | Coefficient | p-value | 95% CI | |
| --- | --- | --- | --- | --- |
| Body fat | 0.002 | 0.888 | -0.020 | 0.023 |
| Age | 0.001 | 0.927 | -0.012 | 0.013 |
| Log Testosterone | -0.035 | 0.725 | -0.232 | 0.162 |
| Constant | 0.831 | 0.431 | -1.241 | 2.904 |
